# Relationship between substance use (tobacco, alcohol, and cannabis) and low academic achievement

**DOI:** 10.1192/j.eurpsy.2023.1371

**Published:** 2023-07-19

**Authors:** E. Gharbi, O. Kaddour, R. Ben Soussia

**Affiliations:** 1 Psychiatric Department, CHU Taher Sfar Mahdia; 2 Faculte de Medecine Monastir, Mahdia, Tunisia

## Abstract

**Introduction:**

Poly use of psychoactive substances (tobacco, alcohol and cannabis) is a major health issue, especially among younger children, mainly adolescents and students. The prevalence of addictive behaviour in Tunisia is clearly increasing. This development attracts the attention of the scholarly society from the point of view of the effects and consequences on the short or long term. Since this is still a taboo subject given the socio-cultural and religious constraints, there are still difficulties in conducting epidemiological investigations on this subject.

**Objectives:**

In this context, we conducted this study with the Tunisian student population during the academic year 2020/2021. The objectives of this work were: To determine the prevalence of the use of the three psychoactive substances (tobacco, alcohol and cannabis) and to evaluate school performance as associated factors of substance use.

**Methods:**

We proceeded to a descriptive and analytical cross-sectional study conducted with a sample of the Tunisian student population during the 2020/2021 academic year (from November 2020 to February 2021).

**Results:**

We included in the analysis 772 students. The average age of the study population was 23.29 3.25. Alcohol was the most reported substance in 35.8% of participants. The prevalence of tobacco use was 32.1% and that of cannabis use was 14.4%. Academic achievement was assessed by the notion of repetition, from which we noted that 81.3% of respondents did not experience repetition. Repetition was statistically significantly associated with the use of all three substances (p < 10-3). The concept of repetition increased the risk of smoking by 2.616 (95% IC95 [1,724-3,969]). The concept of repetition increased the risk of consumption by 2.33 (IC95%[1,522-3,578]). The notion of repetition multiplies the risk of cannabis use by 2,250 (IC95%[1,369-3,699]).

**Conclusions:**

The relationship between the use of psychoactive substances and low academic performance has been explained from different medical and psychological perspectives. It is crucial to recommend the development of support cells in academic institutions as well as to strengthen psychological management especially for people who have experienced a drop in school because they would be people at risk of developing a substance use.

**Disclosure of Interest:**

None Declared

